# Supplementary figures and images for: hsegHMM: hidden Markov model-based allele-specific copy number alteration analysis accounting for hypersegmentation
Source: BMC Bioinformatics. 2018 Nov 14;19:424. doi: 10.1186/s12859-018-2412-y (PMC6236906; doi:10.1186/s12859-018-2412-y)

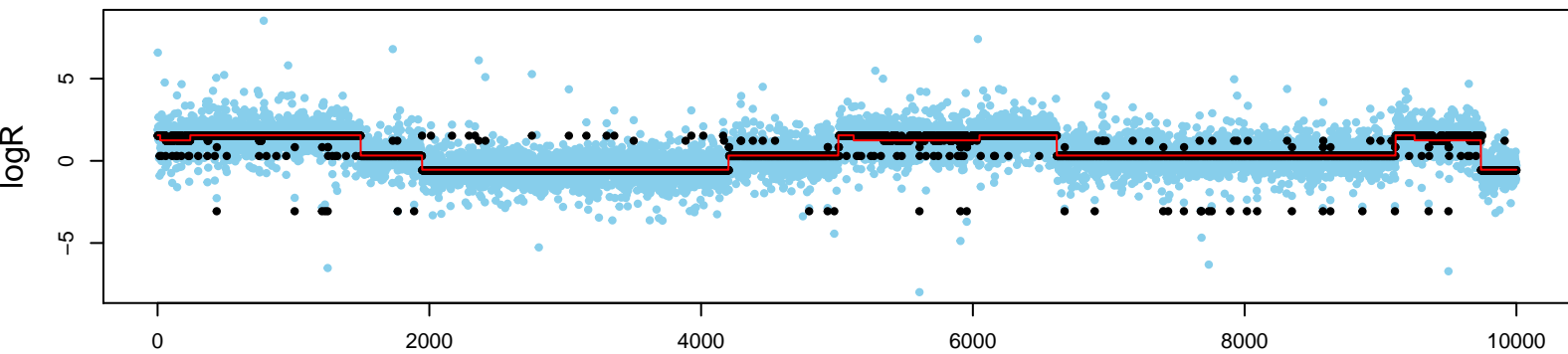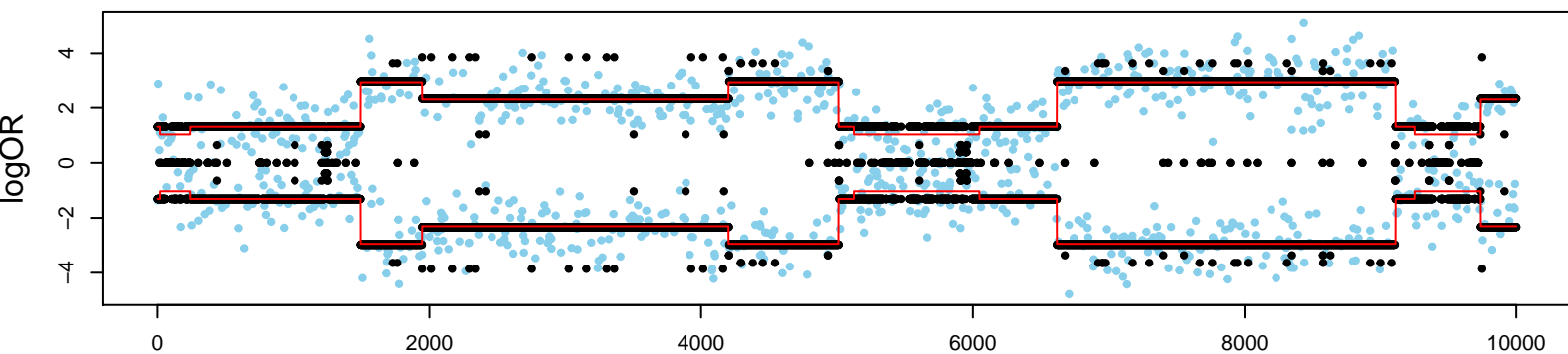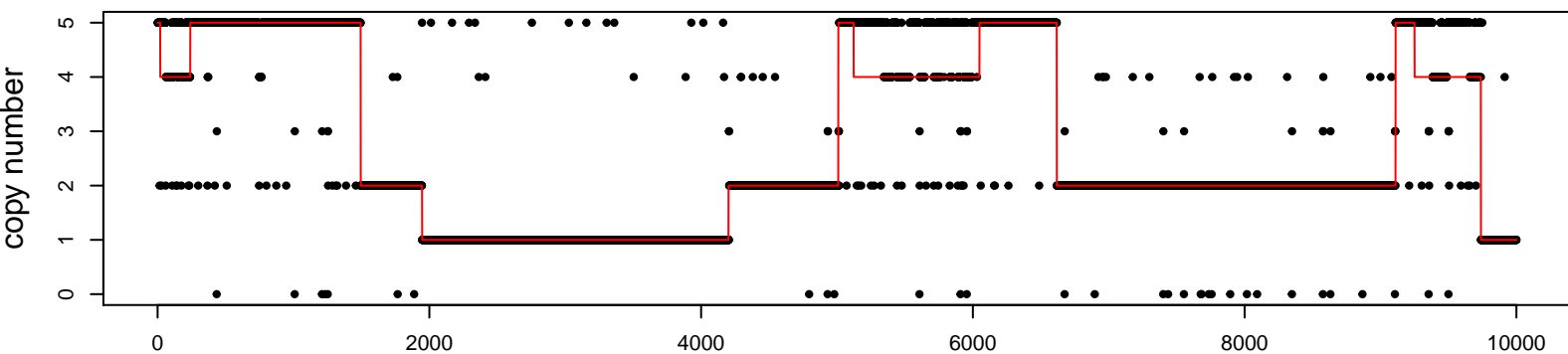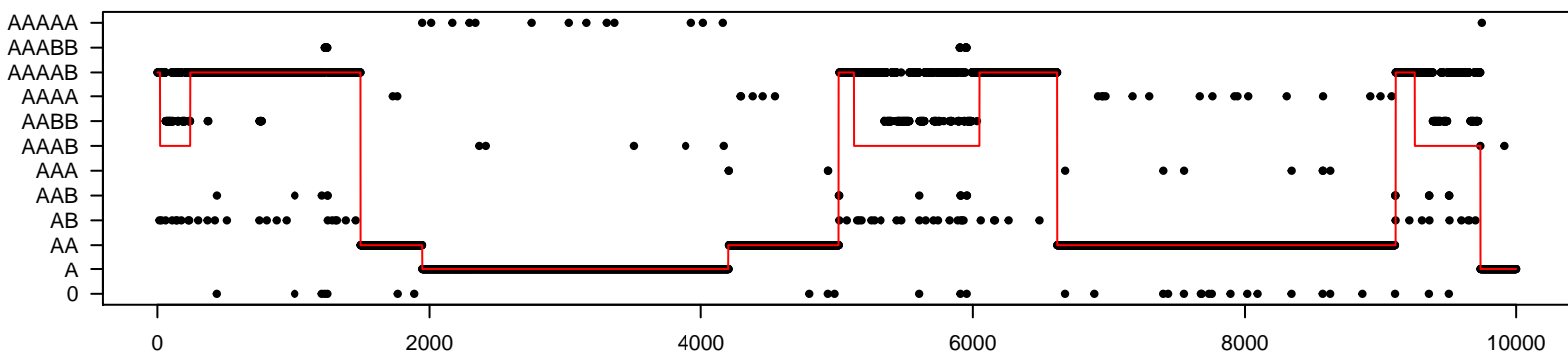

Supplement: Supplementary file 1 — Figure S1. Allele-specific SCNA analysis based on the hsegHMM-N model of a simulated dataset for the simulation study with logR generated from t-distribution. The first two panels show the profiles of logR and logOR over the entire chromosomes; The last two panels indicate estimated copy numbers and genotype for each sequence over the entire chromosomes. (PDF 2416 kb) [file 12859_2018_2412_MOESM1_ESM.pdf]

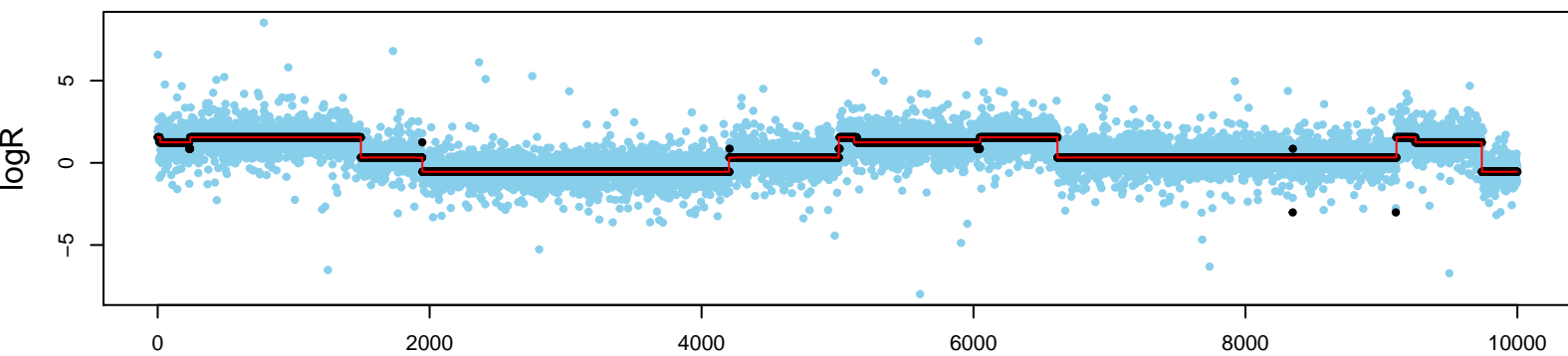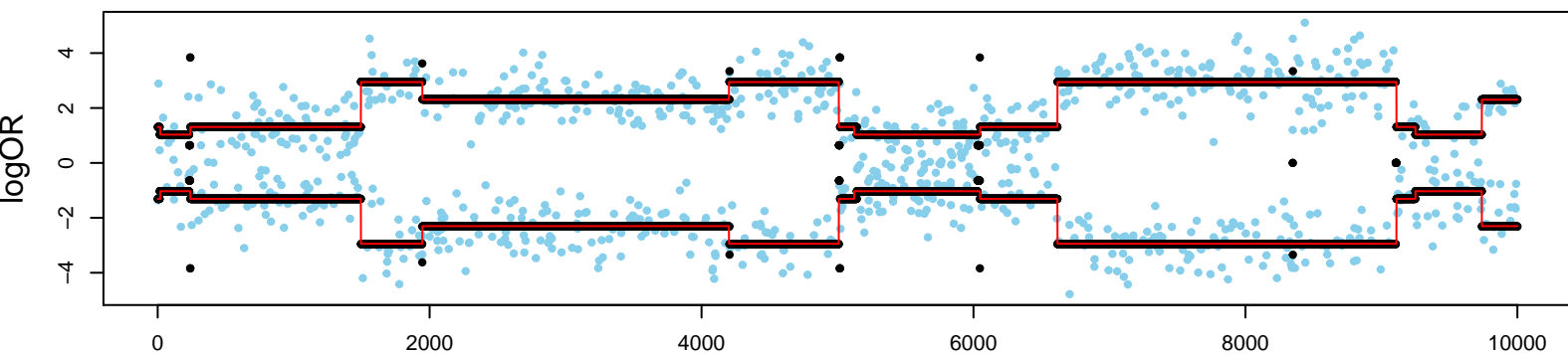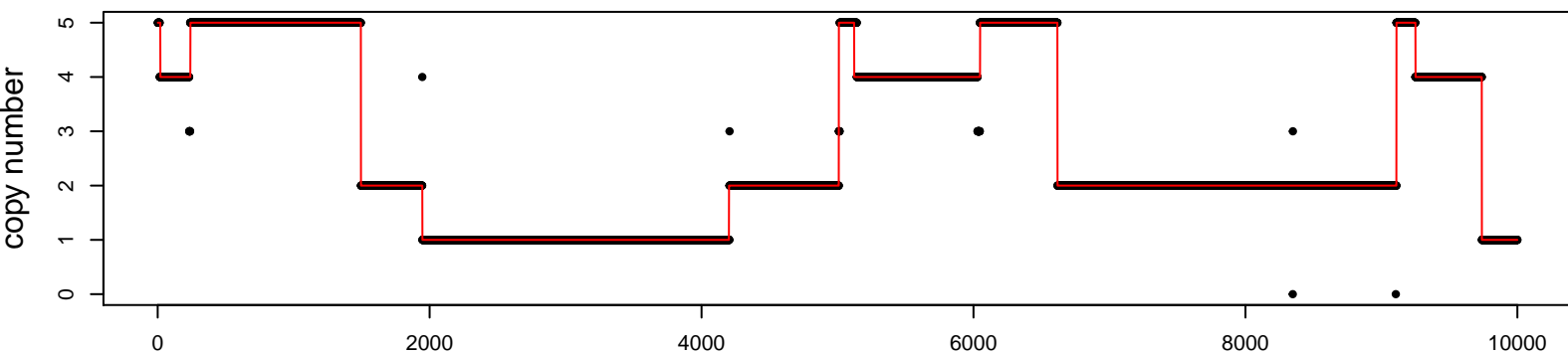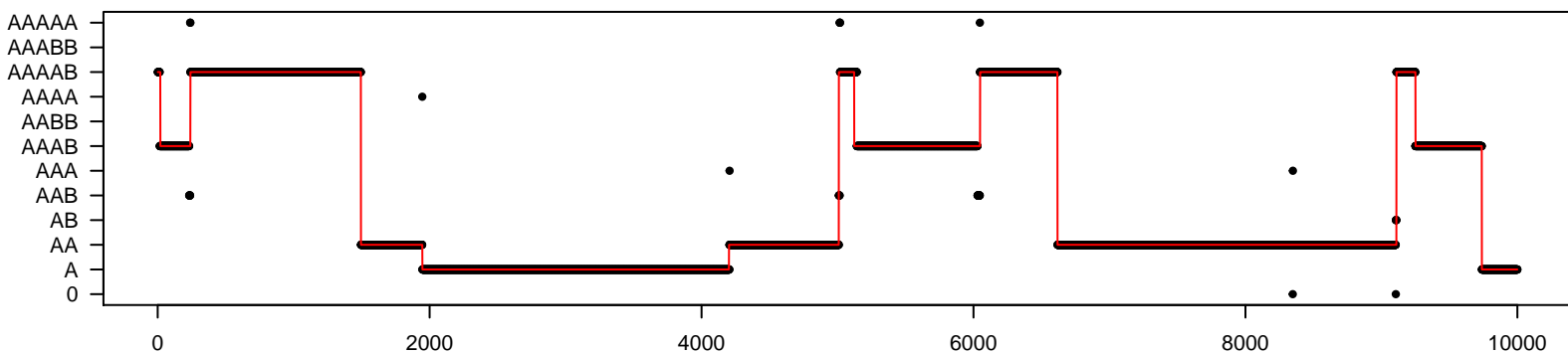

Supplement: Supplementary file 2 — Figure S2. Allele-specific SCNA analysis based on the hsegHMM-T model of a simulated dataset for the simulation study with logR generated from t-distribution. The first two panels show the profiles of logR and logOR over the entire chromosomes; The last two panels indicate estimated copy numbers and genotype for each sequence over the entire chromosomes. (PDF 2389 kb) [file 12859_2018_2412_MOESM2_ESM.pdf]

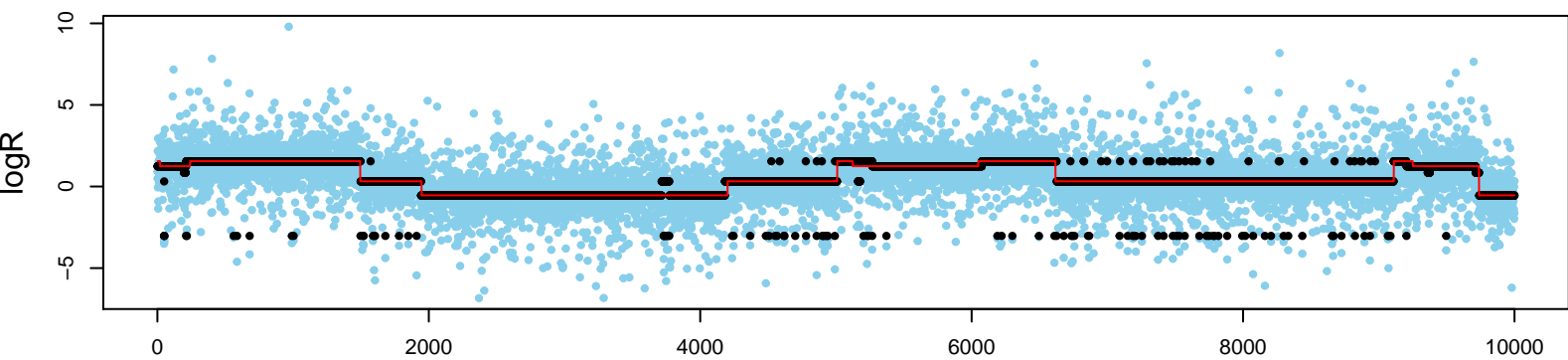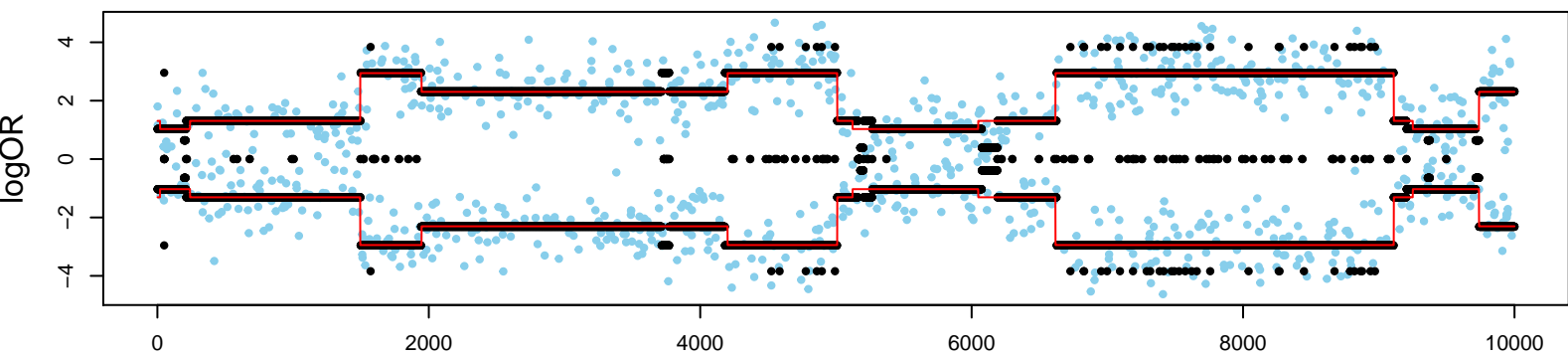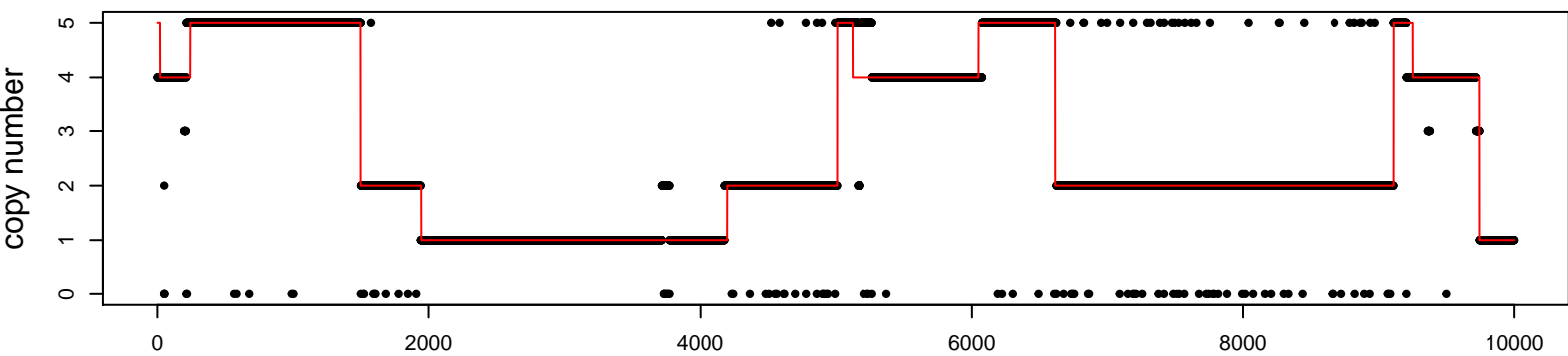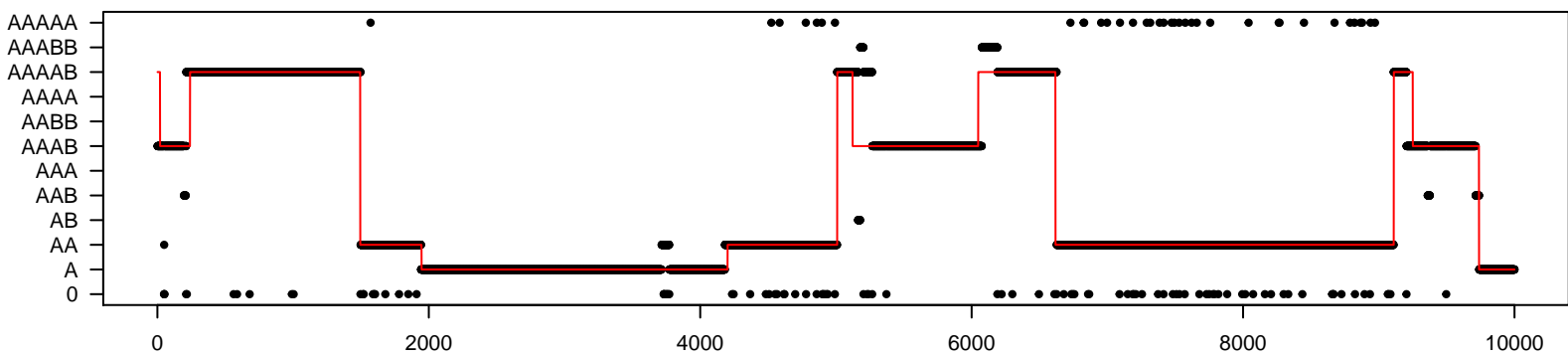

Supplement: Supplementary file 3 — Figure S3. Allele-specific SCNA analysis based on the hsegHMM-N model of a simulated dataset for the simulation study with logR generated from normal-mixture distribution. The first two panels show the profiles of logR and logOR over the entire chromosomes; The last two panels indicate estimated copy numbers and genotype for each sequence over the entire chromosomes. (PDF 2407 kb) [file 12859_2018_2412_MOESM3_ESM.pdf]

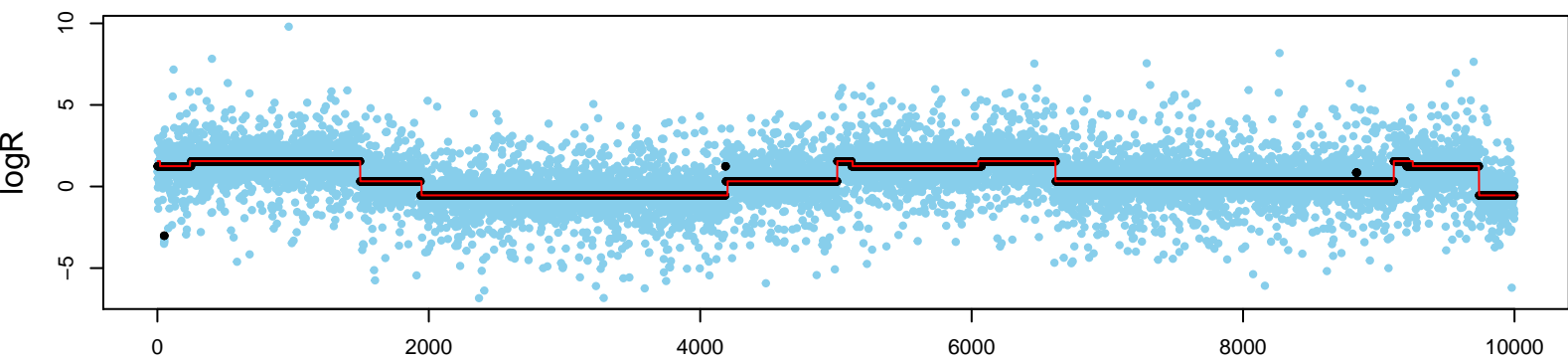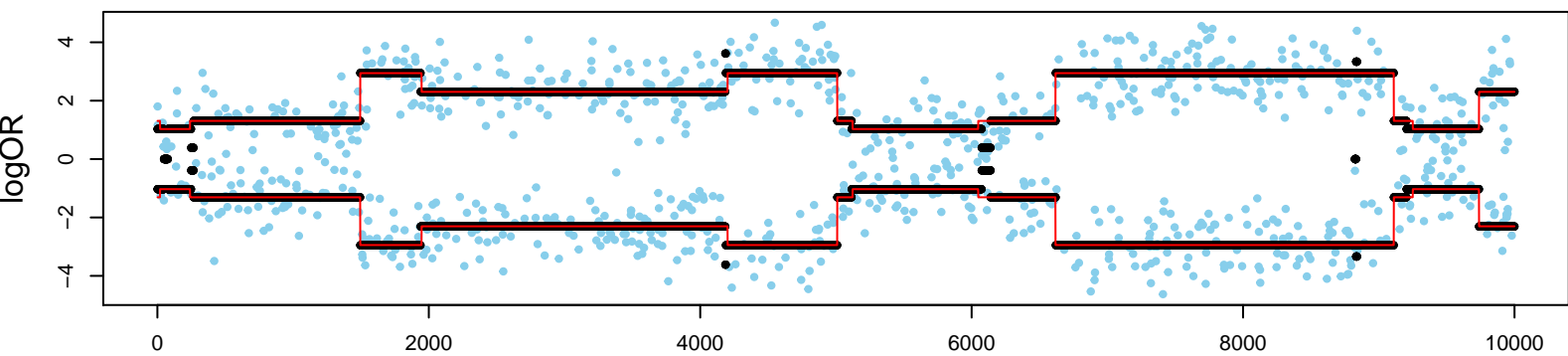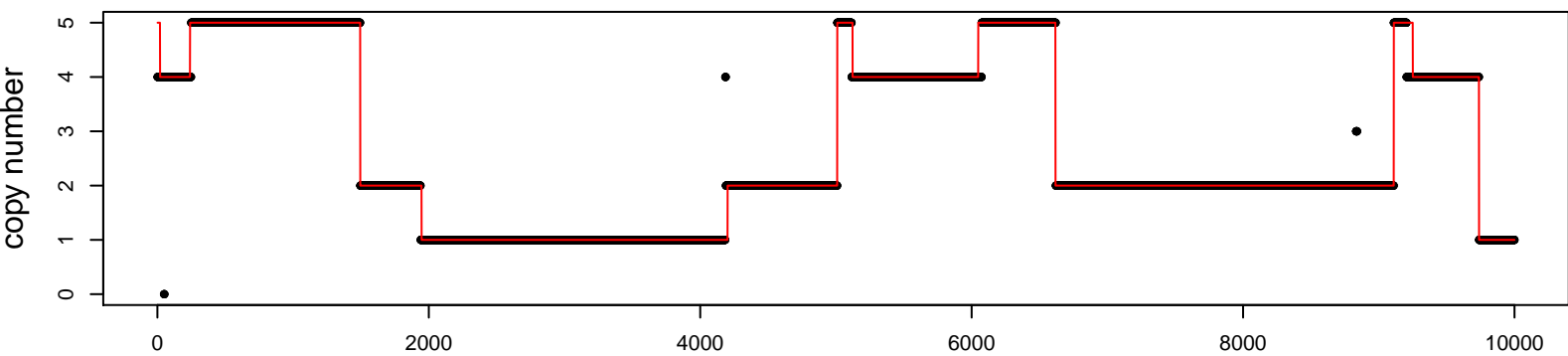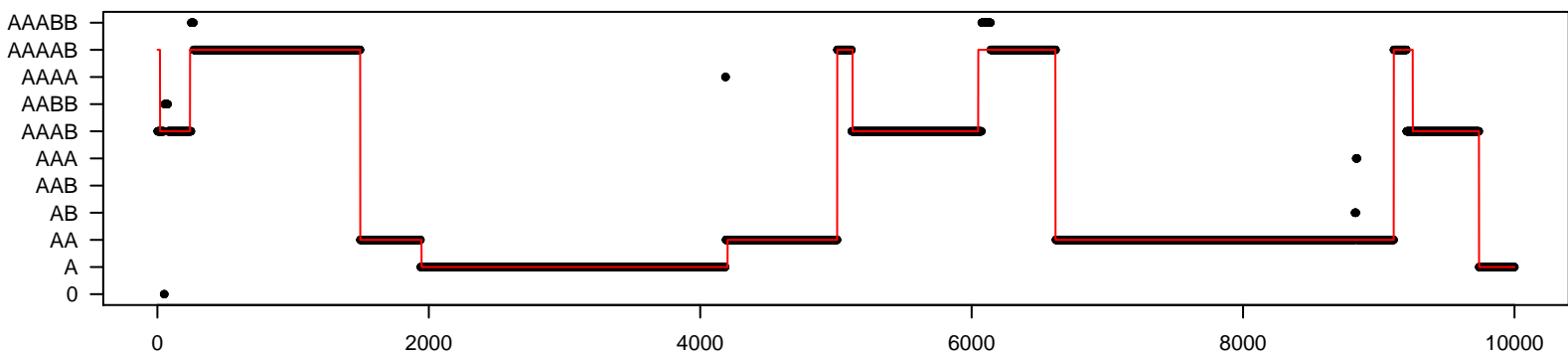

Supplement: Supplementary file 4 — Figure S4. Allele-specific SCNA analysis based on the hsegHMM-T model of a simulated dataset for the simulation study with logR generated from normal-mixture distribution. The first two panels show the profiles of logR and logOR over the entire chromosomes; The last two panels indicate estimated copy numbers and genotype for each sequence over the entire chromosomes. (PDF 2387 kb) [file 12859_2018_2412_MOESM4_ESM.pdf]

Probability of Identification

AA

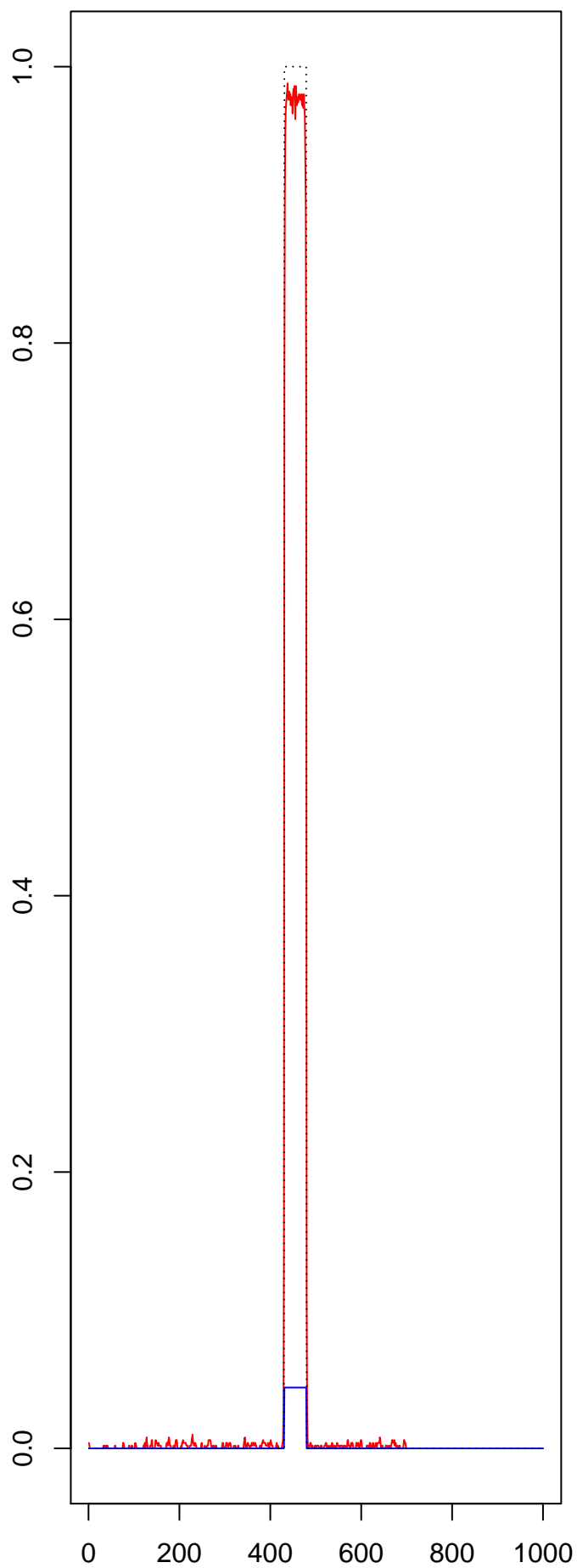

Probability of Identification

AA

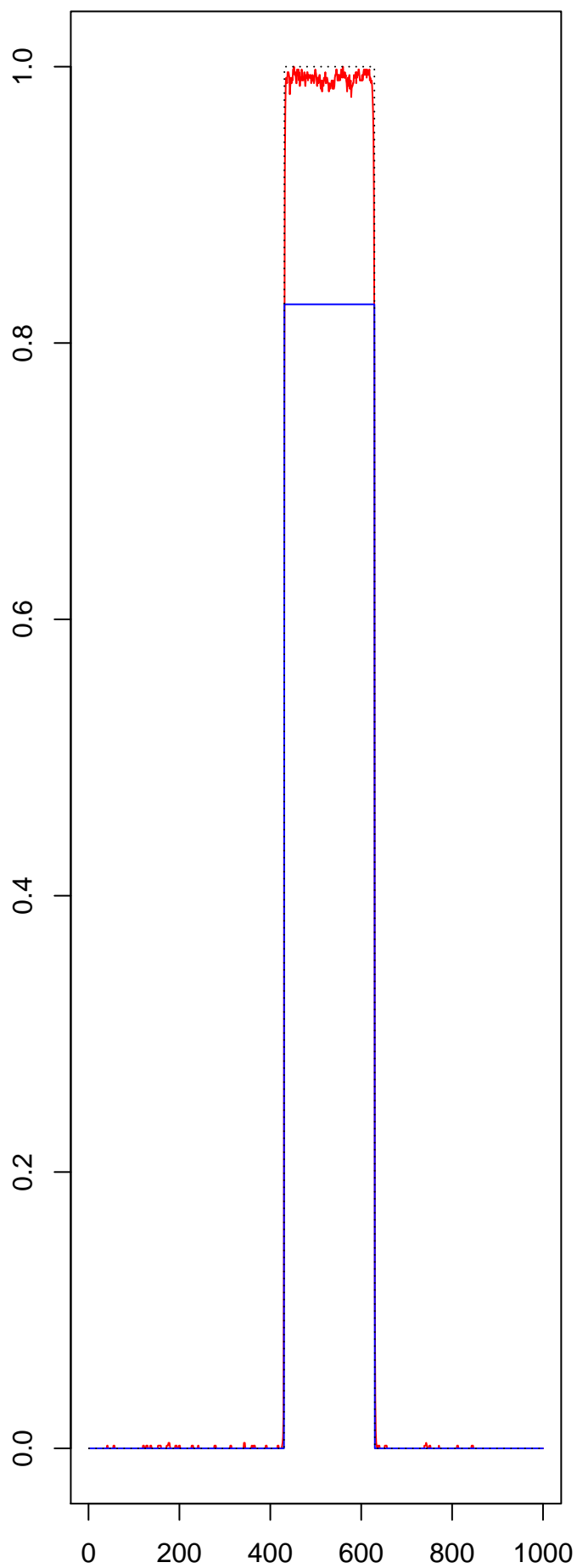

Supplement: Supplementary file 6 — Figure S6. Probability of identification for a region generated from a non-standard beta-based read depths. The red line and blue line represents hsegHMM-T and FACETS; The black dotted line is the true one. (PDF 13 kb) [file 12859_2018_2412_MOESM6_ESM.pdf]

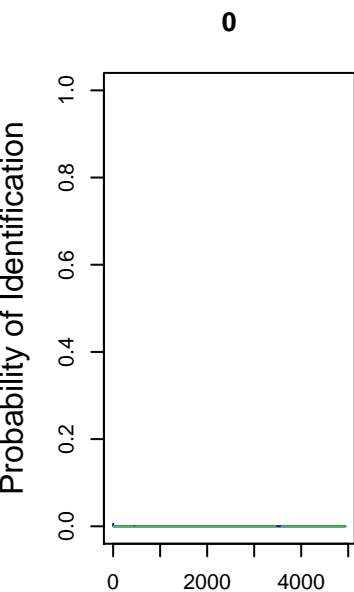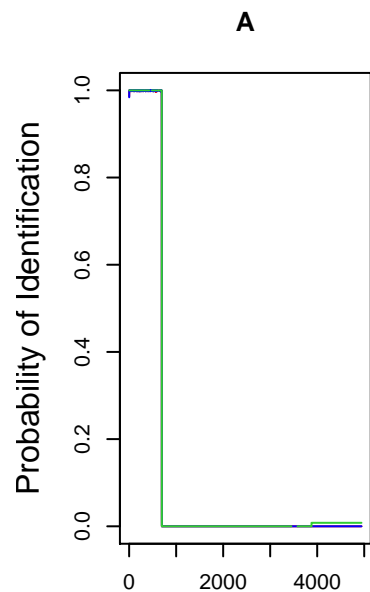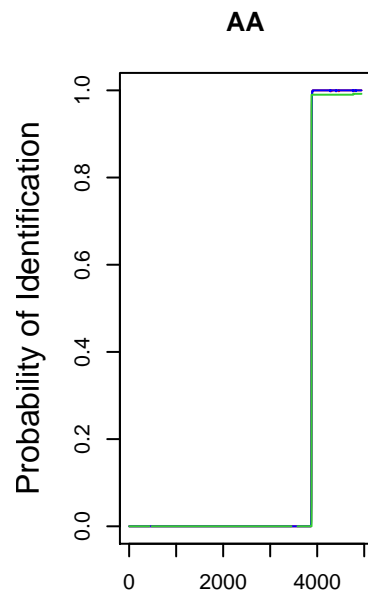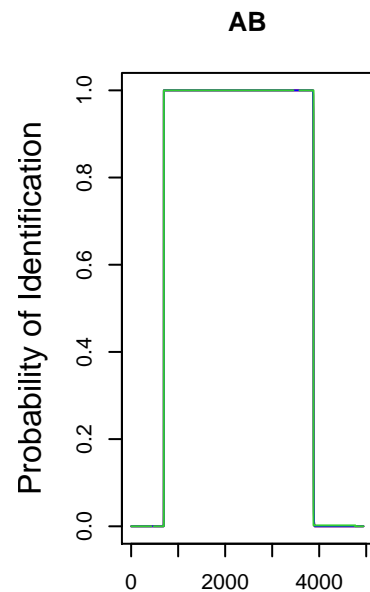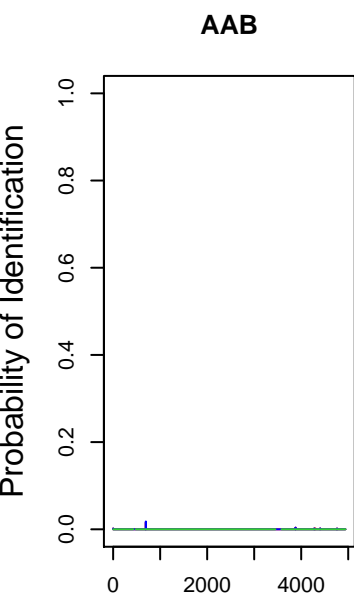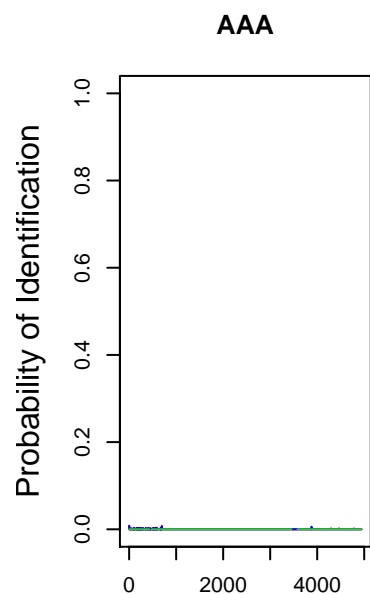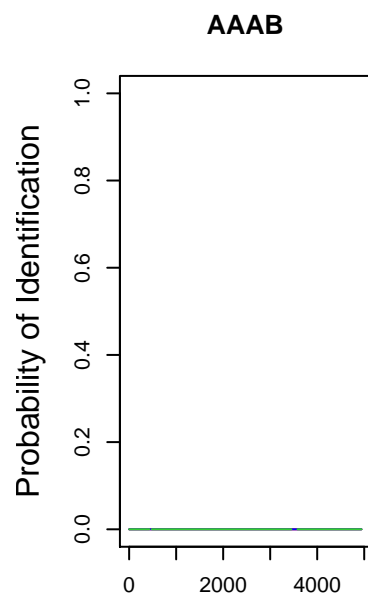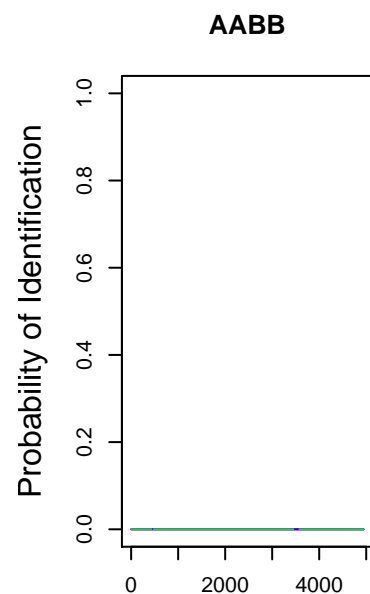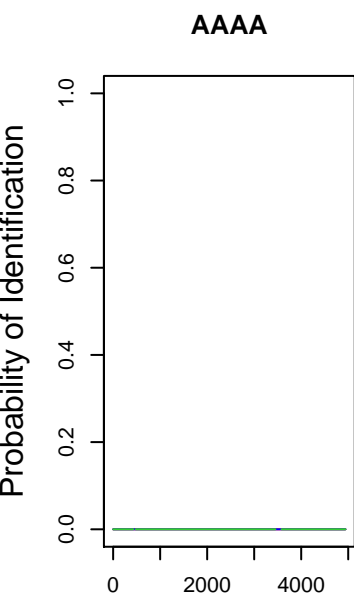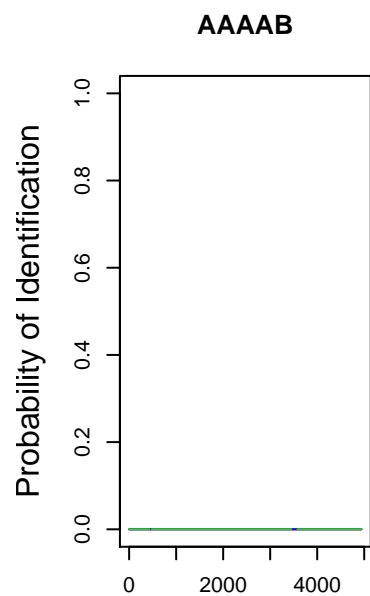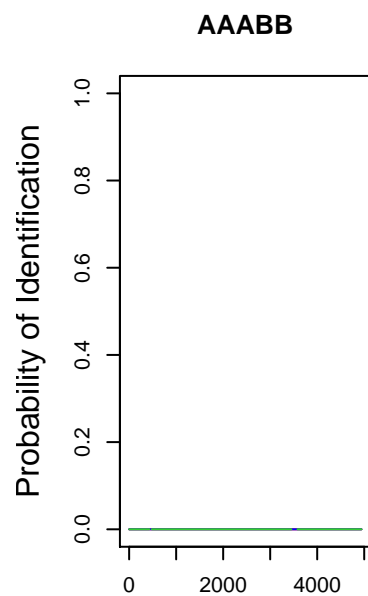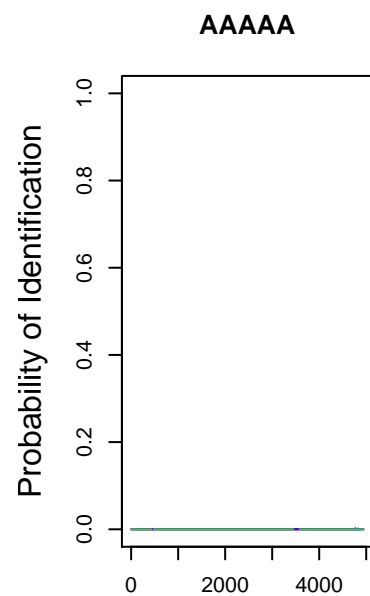

Supplement: Supplementary file 7 — Figure S7. Probability of identification with the half size of read counts and depths from Figure 6 green lines, blue lines, and red lines indicate the probabilities of identification based on the FACETS, hsegHMM-N, and hsegHMM-T models, respectively; Each dataset consists of 4,942 observations of logR and logOR. (PDF 437 kb) [file 12859_2018_2412_MOESM7_ESM.pdf]

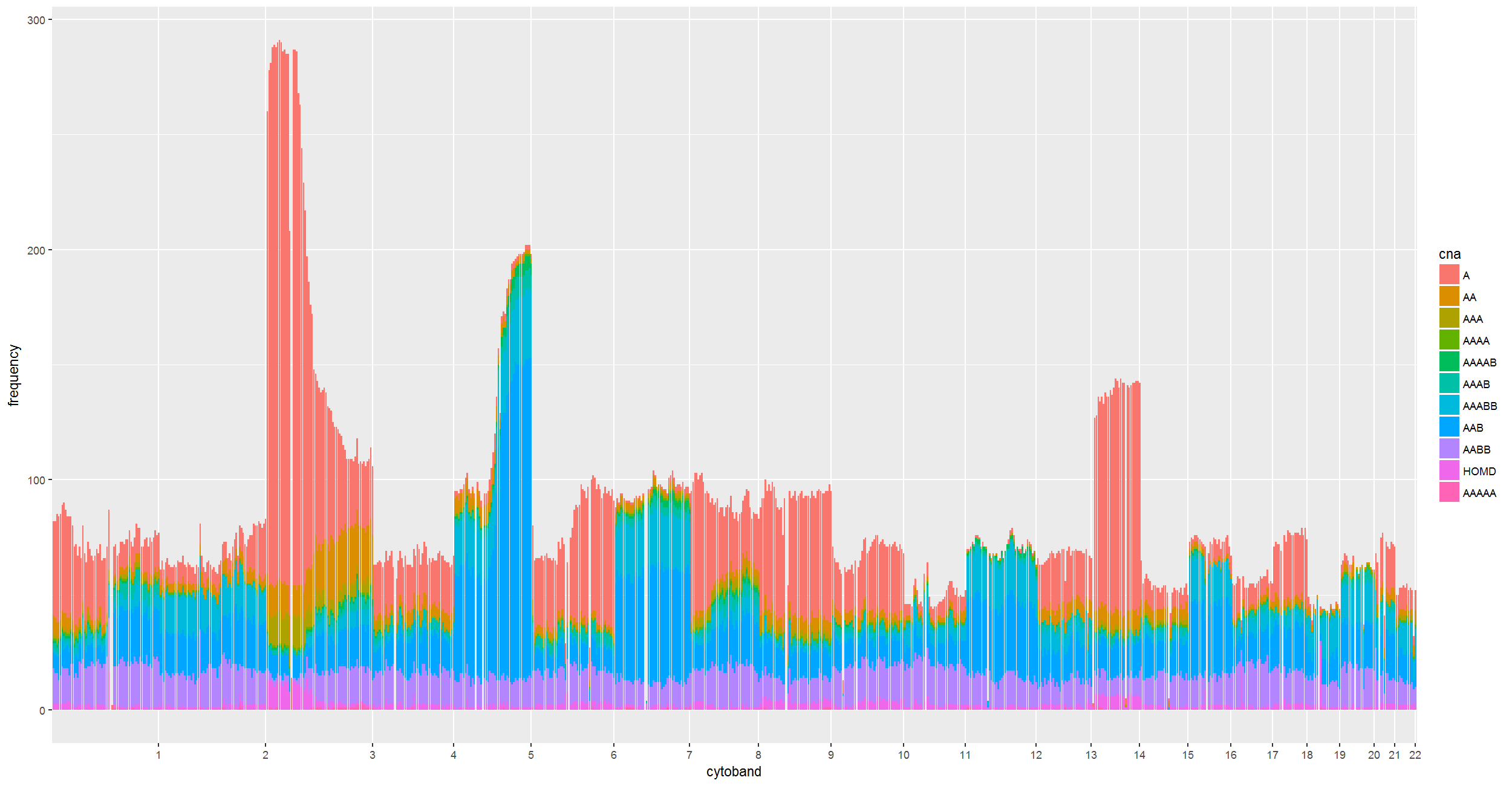

Supplement: Supplementary file 9 — Figure S9. Frequency of Allele-specific SCNA events based on cytobands across all the chromosomes for 316 samples from TCGA "HOMD" indicates homozygous deletion state. (TIF 9522 kb) [file 12859_2018_2412_MOESM9_ESM.tif]
