# Supplementary material for: hsegHMM: hidden Markov model-based allele-specific copy number alteration analysis accounting for hypersegmentation
Source: BMC Bioinformatics. 2018 Nov 14;19:424. doi: 10.1186/s12859-018-2412-y (PMC6236906; doi:10.1186/s12859-018-2412-y)
Supplement: Supplementary file 5 — Figure S5. Probability of identification for different sizes of purity with logR generated from the normal-mixture distribution. The red lines, green lines, and gold lines represent the high (α=0.7), medium (α=0.5), and low (α=0.3) purity cases. All the results are conducted with hsegHMM-T. (PDF 898 kb) [file 12859_2018_2412_MOESM5_ESM.pdf]

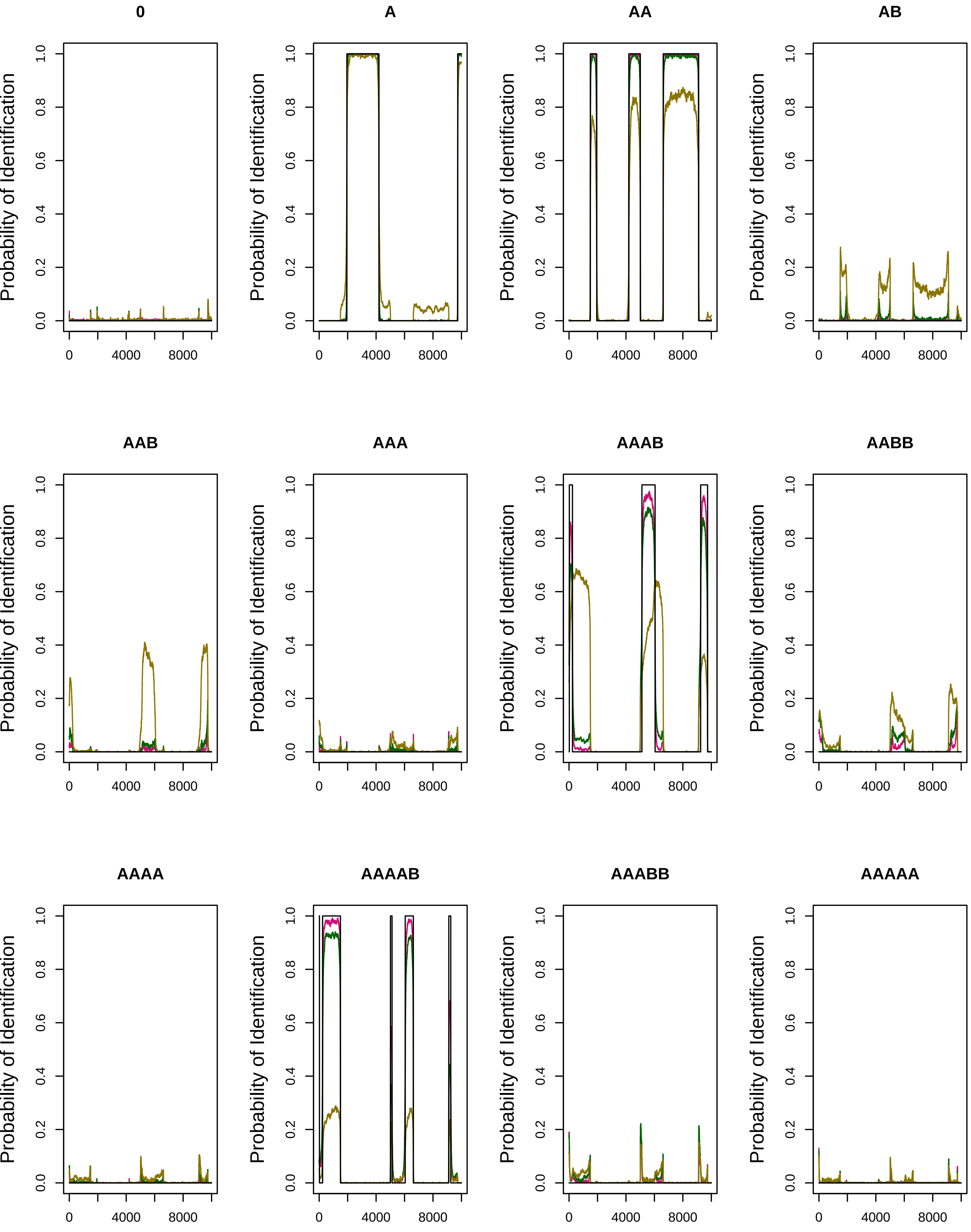
0
A
AA
AB
AAB
AAA
AAAB
AABB
AAAA
AAAAB
AAABB
AAAAA
Probability of Identification
0.0
0.2
0.4
0.6
0.8
1.0
0
4000
8000
