# Supplementary material for: hsegHMM: hidden Markov model-based allele-specific copy number alteration analysis accounting for hypersegmentation
Source: BMC Bioinformatics. 2018 Nov 14;19:424. doi: 10.1186/s12859-018-2412-y (PMC6236906; doi:10.1186/s12859-018-2412-y)
Supplement: Supplementary file 8 — Figure S8. Probability of identification with the double size of read counts and depths from Figure 6 green lines, blue lines, and red lines indicate the probabilities of identification based on the FACETS, hsegHMM-N, and hsegHMM-T models, respectively; Each dataset consists of 4,942 observations of logR and logOR. (PDF 436 kb) [file 12859_2018_2412_MOESM8_ESM.pdf]

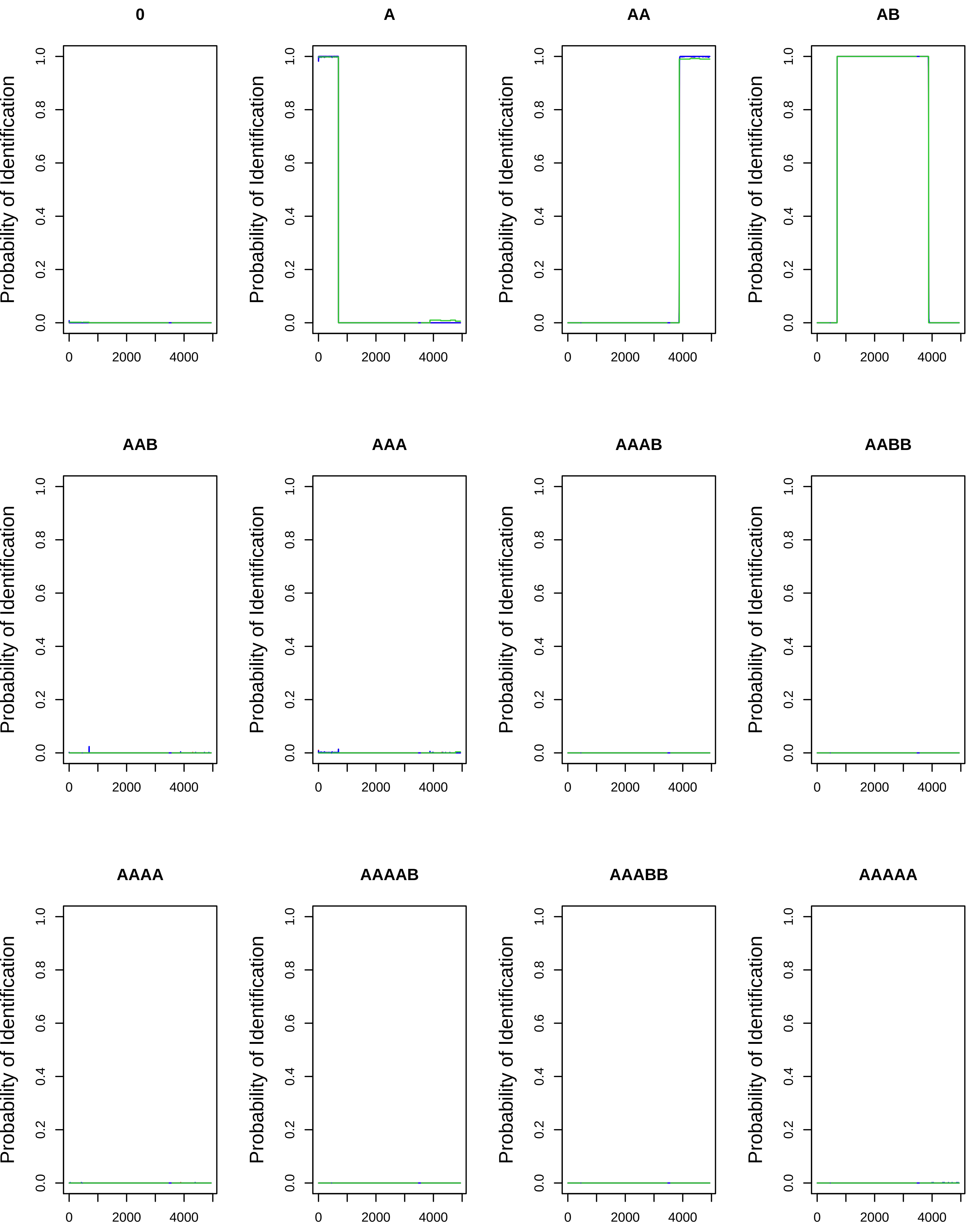
0
A
AA
AB
AAB
AAA
AAAB
AABB
AAAA
AAAAB
AAABB
AAAAA
Probability of Identification
0.0
0.2
0.4
0.6
0.8
1.0
0
2000
4000
